# Functional connectivity correlates of reaction time variability in treatment-resistant major depression

**DOI:** 10.1017/S0033291726104899

**Published:** 2026-07-07

**Authors:** Paul Michael Briley, Lucy Webster, Beth Hall, Linda Davison, Peter Gallagher, Stefan Pszczolkowski, Sudheer Lankappa, Dorothee P. Auer, Peter F. Liddle, R. Hamish McAllister-Williams, Richard Morriss

**Affiliations:** 1 Mental Health and Clinical Neurosciences, University of Nottingham School of Medicine, UK; 2 Nottinghamshire Healthcare NHS Foundation Trust, UK; 3 NIHR Nottingham Biomedical Research Centre, UK; 4 Translational and Clinical Research Institute, Newcastle University, UK; 5 Northern Centre for Mood Disorders, Newcastle University, UK; 6 Cumbria, Northumberland, Tyne & Wear NHS Foundation Trust, UK; 7 Sir Peter Mansfield Imaging Centre, University of Nottingham School of Medicine, UK; 8 NIHR Newcastle Biomedical Research Centre, UK

**Keywords:** brain connectivity, cognitive impairment, depression, executive control network (ECN), intra-individual variability, major depressive disorder (MDD), salience network (SN), social and occupational functioning, sustained attention, functional magnetic resonance imaging (fMRI)

## Abstract

**Background:**

Cognitive difficulties, including problems with attention and executive processing, are common in major depressive disorder (MDD), and strongly predict psychosocial and occupational functioning. Impairment in sustained attention contributes to increased intra-individual variability (IIV) in reaction times observed during cognitive tasks. Understanding brain network changes associated with IIV could guide novel neuromodulation strategies targeting cognitive difficulties.

**Methods:**

We analyzed baseline resting-state fMRI data from 209 patients with moderate-to-severe treatment-resistant MDD who participated in the BRIGhTMIND neuromodulation trial. Following a preregistered analytic protocol, we examined associations between: functional connectivity across three core brain networks (executive control, ECN; default mode, DMN; and salience network, SN); components of IIV derived from a choice reaction time task (using a three-parameter ex-Gaussian model); and functioning.

**Results:**

Greater IIV was linked to increased ECN-DMN functional connectivity. The ECN supports top-down control and externally directed cognition, while the DMN supports internal mentation and rumination. ECN-DMN connectivity was modulated by the SN, which prioritizes salient internal and external stimuli. Higher SN-ECN connectivity was associated with lower ECN-DMN connectivity and with faster mean reaction times. Both IIV and mean reaction time predicted functioning, with poorer functioning related to a slowed and inflexible response pattern.

**Conclusions:**

Distinct components of reaction time variability are associated with specific patterns of brain network connectivity, largely independent of mood severity. Connectivity between the salience and executive control networks may represent a promising target for neuromodulation interventions focused on cognitive deficits in MDD.

## Introduction

As many as 85–94% of people with major depressive disorder (MDD) report cognitive difficulties during depressive episodes (Conradi, Ormel, & De Jonge, 2011). Neurocognitive deficits are wide ranging (Hammar, Ronold, & Rekkedal, 2022; McDermott & Ebmeier, 2009; Perini et al., 2019; Rock, Roiser, Riedel, & Blackwell, 2014), often persist during periods of euthymia (Huang, 2009; Semkovska et al., 2019), and strongly predict psychosocial and occupational dysfunction (Knight, Air, & Baune, 2018; Matcham et al., 2023; McIntyre et al., 2013). While broad in nature, the cognitive deficits in MDD are driven by impairments in attention, executive function, working memory, and psychomotor speed (Schandorff et al., 2025). Understanding the neural basis of attentional deficits could guide the development of augmentation approaches to better target cognitive difficulties in MDD (Colwell et al., 2022; Zuckerman et al., 2018). If there are changes at the brain network level, noninvasive brain stimulation (neuromodulation) techniques that modify brain networks (To, De Ridder, Hart, & Vanneste, 2018), such as repetitive transcranial magnetic stimulation (rTMS), could play an important role.

The triple network model hypothesizes that abnormal connectivity within and between three core brain networks underpins cognitive deficits, emotional dysfunction, and other key symptoms of MDD (Menon, 2019). These are: the executive control network (ECN) – involved in working memory, attention, and goal-directed behavior (Dosenbach et al., 2008); the default mode network (DMN) – involved in internally-directed mental activity, including rumination (Raichle, 2015); and the salience network (SN) – involved in identifying the most important internal and external stimuli or processes (Seeley et al., 2007). One of the most robust findings in the literature on MDD is increased direct coupling of network activity (functional connectivity, ‘FC’) between the ECN and DMN (Kaiser, Andrews-Hanna, Wager, & Pizzagalli, 2015). Based on findings that *lesser* coupling of activity (i.e. lower FC) between the ECN and DMN is associated with *better* cognitive performance in healthy volunteers (Keller et al., 2013; Kelly et al., 2008), it might be expected that stronger ECN-DMN connectivity underlies cognitive impairments in MDD. Such abnormal connectivity might represent intrusion of DMN-related internal world processing or rumination on ECN-related attention and task performance (Viola et al., 2025). Since the SN plays an important role in regulating the relative activities of the ECN and DMN (Goulden et al., 2014; Sridharan, Levitin, & Menon, 2008), SN-ECN or SN-DMN connectivity might represent a more proximal cause of cognitive impairments. A recent meta-analysis demonstrated hypoconnectivity between the SN and both the ECN and DMN across psychiatric disorders (Sha, Wager, Mechelli, & He, 2019).

Impairment in sustained attention is reflected in increased intra-individual variability (IIV) in response times during cognitive tasks, due to trial-by-trial fluctuations in performance. IIV is considered a sensitive measure of attention and cognitive control (Klein et al., 2006) and increased IIV may be a core feature of the cognitive impairment seen in mood disorders (Gallagher et al., 2015; Viola et al., 2025). The distribution of reaction times across trials of an attentional task for a given individual can be well-characterized with an exponentially modified Gaussian (‘ex-Gaussian’) distribution, which consists of (i) a Gaussian component – with parameters ‘MU’, equivalent to the mean of the Gaussian component reflecting the average reaction time of the participant, and ‘SIGMA’, equivalent to the standard deviation of the Gaussian component reflecting reaction time variability; and (ii) an exponential component – with parameter ‘TAU’, which describes unusually slow responses or ‘attentional lapses’ (Gallagher et al., 2015; Schmiedek et al., 2007; Silvia et al., 2020). It may be that each component is differentially related to brain connectivity changes in MDD.

Here, we use baseline brain imaging and cognitive task data from 209 participants with treatment-resistant, moderate-to-severe MDD who took part in the BRIGhTMIND clinical trial of rTMS (Morriss et al., 2024) to test four hypotheses on relationships between brain connectivity and IIV, based on the above rationale. Importantly, given concerns around analytic flexibility in imaging studies (Briley et al., 2024a; Kang et al., 2024; Marek & Laumann, 2024), these hypotheses, and associated analysis steps, were prespecified in a published analytic protocol, before connecting the imaging and cognitive datasets (Briley et al., 2024b). These are: (1) ECN-DMN FC will be positively correlated with IIV; (2) ECN-DMN FC will be positively correlated with subjective cognitive difficulties (self-reported difficulties are highly correlated with mood symptoms, but poorly correlated with objective measures, suggesting they capture different aspects of impairment; Serra-Blasco et al., 2019); (3) ECN-DMN FC will be predicted by FC between the SN and the ECN and DMN (reflecting the modulatory or network-switching role of the SN); and (4) IIV will be related to occupational and social functioning in MDD (Lam, Kennedy, McIntyre, & Khullar, 2014). After examining the above hypotheses, we conduct a path analysis to synthesize our findings.

## Materials and methods

We analyzed baseline resting-state fMRI, THINC-it cognitive task, and demographic/clinical questionnaire data from the BRIGhTMIND trial (Morriss et al., 2020, 2024). We tested four hypotheses (plus related exploratory analyses) following a prespecified analytic protocol (Briley et al., 2024b), which was built on protocols for processing the fMRI (Briley et al., 2022; Pszczolkowski et al., 2022) and THINC-it (Hall et al., 2024) datasets. We then synthesize these findings using path analysis.

### Participants

From 255 trial participants, 209 met prespecified data availability and quality-control (QC) criteria for inclusion in these analyses (Supplementary Figure S1). Participants were recruited at four trial sites (Nottingham, Northampton, Newcastle, and London) and scanned at three MRI sites (Northampton participants were scanned at Nottingham). The later-added Oldham site and Northampton participants scanned with the Ingenia MRI scanner (see MRI measurements) were omitted as per the analysis protocol due to the small number of participants. Reasons for exclusion were: excluded site or scanner (*N* = 8), imaging QC failure (*N* = 15), THINC-it data not available (*N* = 4), and THINC-it QC failure (*N* = 19). Demographic and clinical characteristics did not significantly differ between included and excluded participants (Table 1).

**Table 1.** Comparison of demographic and clinical variables between included/excluded participants for the cognition-imaging analyses Table 1. long description.

Characteristic	Included (*n* = 209)	Excluded (*n* = 46)	Comparison
Age in years – mean (SD)	44.4 (14.0)	43.4 (14.4)	*t*(253) = 0.444, *p* = 0.658
Gender – *n* (%)			*χ^2^* (1)=0.349, *p* = 0.555
Men	99 (47.4%)	24 (47.8%)	
Women	110 (52.6%)	22 (52.2%)	
Ethnicity – *n* (%)			*χ^2^* (1)=0.072, *p* = 0.789
White British	176 (84.2%)	38 (82.6%)	
Other	33 (15.8%)	8 (17.4%)	
MGH treatment resistance category – *n* (%)			*χ^2^* (2)=0.850, *p* = 0.654
Low (2–3.5)	69 (33.0%)	18 (39.1%)	
Medium (4–6)	62 (29.7%)	11 (23.9%)	
High (6.5+)	78 (37.3%)	17 (37.0)	
HAMD total – mean (SD)	23.3 (4.8)	24.4 (4.7)	*t*(253) = 1.407, *p* = 0.161
GAD–7 total – mean (SD)	13.3 (4.7)	13.1 (4.5)	*t*(253) = 0.287, *p* = 0.775
WSAS total – mean (SD)	28.1 (7.6)	29.2 (6.2)	*t*(253) = 0.960, *p* = 0.338

*Note*: Statistics are *t*-tests for continuous variables and Chi-square tests for categorical variables.

GAD-7, Generalized Anxiety Disorder scale; HAMD, Hamilton Depression Rating Scale; MGH, Massachusetts General Hospital treatment resistance scale; WSAS, Work and Social Adjustment Scale.

Trial participants were adults ≥18 years with current MDD (DSM-5), of at least moderate severity (Hamilton Depression Rating Scale, HAMD ≥16), and treatment resistance (Massachusetts General Hospital Scale, MGH ≥ 2). Exclusion criteria included bipolar disorder, depression secondary to other mental disorders, current suicidality, substance abuse/dependence, neurological conditions, recent anticonvulsant use, daily benzodiazepine use >5 mg diazepam-equivalent, pregnancy, and standard MRI contraindications.

The authors assert that all procedures contributing to this work comply with the ethical standards of the relevant national and institutional committees on human experimentation and with the Helsinki Declaration of 1975, as revised in 2008.

### Questionnaire and cognitive measures

At baseline, participants completed self-reported measures, including the GAD-7 (anxiety severity; Spitzer, Kroenke, Williams, & Löwe, 2006) and the WSAS (social/occupational functioning; Mundt, Marks, Shear, & Greist, 2002). The observer-rated HAMD was also completed to assess depression severity (Hamilton, 1967). Cognitive testing used the THINC-it battery of tests (McIntyre et al., 2017) presented on a touch screen tablet. Analyses of the THINC-it dataset alone are reported in Morriss et al. (2025). This is the first manuscript to combine the THINC-it and imaging datasets. Analyses here focus on the Choice Reaction Time (CRT) task of sustained attention, and the five-item PDQ-5-D measure of subjective cognitive difficulties (findings for the other tasks are in Supplementary Material 1). The CRT presents a series of arrows with a variable inter-trial interval. Participants are asked to press a key as fast as they can to indicate the direction to which each arrow points (left or right). Forty trials are collected per run, and the task was run twice. The distribution of reaction times (RTs) across both runs for correctly-responded-to trials was fitted with an ex-Gaussian distribution (Gallagher et al., 2015; Schmiedek et al., 2007).

Quality control: Any task was excluded if derived variables >4 SD from the mean across participants (Dalby et al., 2022), CRT was excluded if only one run was completed or if ex-Gaussian fitting failed, and the participant was removed if <3 objective THINC-it tasks met QC criteria. As per the prepublished protocol, due to marked right skew in the IIV variables, the natural logarithm transform was applied to MU and SIGMA values, and the cube root transform to TAU values (this brought skewness and kurtosis for these variables within ±1). All analyses were conducted on the transformed variables.

### MRI measurements

Baseline 3T MRI occurred within 2 weeks of the baseline assessment and before treatment randomization. Scanners were GE Discovery MR750/Philips Ingenia/GE Premier (Nottingham), Philips Achieva dStream (Newcastle), and Philips Prisma (London). Northampton participants were scanned at Nottingham using the GE Discovery MR750. T1-weighted images were collected, as well as 8 minutes of eyes-open resting-state (fixation cross) fMRI (gradient echo EPI sequence, 240 volumes, TR/TE 2 s/32 ms, FA 77°, 3 mm^3^ voxels, FOV 192 mm^2^, 35 slices with slice gap 0.5 mm, interleaved bottom/up, and posterior-to-anterior phase encoding). Additional modalities (diffusion tensor, arterial spin labeling, and magnetic resonance spectroscopy) were acquired in some cases; these will be reported elsewhere.

Quality control: T1 structural images with whole head coverage without significant incidental findings or appreciable image artefacts; BOLD images with mean and maximum framewise displacement below 1 and 3 mm, respectively. Participants were excluded with outlying DVARS_STD (third quartile plus 1.5 times interquartile range; standardized measure of image intensity change between consecutive time points; MRIQC toolbox; Esteban et al., 2017), or outlying SNR or TSNR (first quartile minus 1.5 times interquartile range; signal-to-noise and temporal signal-to-noise ratios).

### MRI processing

Structural and resting-state data were preprocessed using the Nottingham BRC pipeline (v1.5.5; github.com/SPMIC-UoN/BRC_Pipeline), which integrates SPM12, FSL, and FreeSurfer tools. Structural processing comprised bias-field correction, brain extraction, tissue segmentation, and nonlinear registration to MNI152 space. Whole brain, gray matter (GM), cerebrospinal fluid (CSF), and white matter (WM) masks were extracted and warped to standard space. WM and CSF images were binarized at 99% tissue-probability threshold, and GM images at 25%. Functional processing comprised distortion correction, motion correction, intensity normalization, slice-timing correction, spatial smoothing (5-mm FWHM), ICA-AROMA physiological noise removal, temporal filtering (0.01 Hz high-pass), and registration to standard space. Mean WM and CSF time courses were regressed from voxel time series; in exploratory analyses, the whole-brain mean signal was also regressed out of time series (global signal regression).

**Figure 1.**
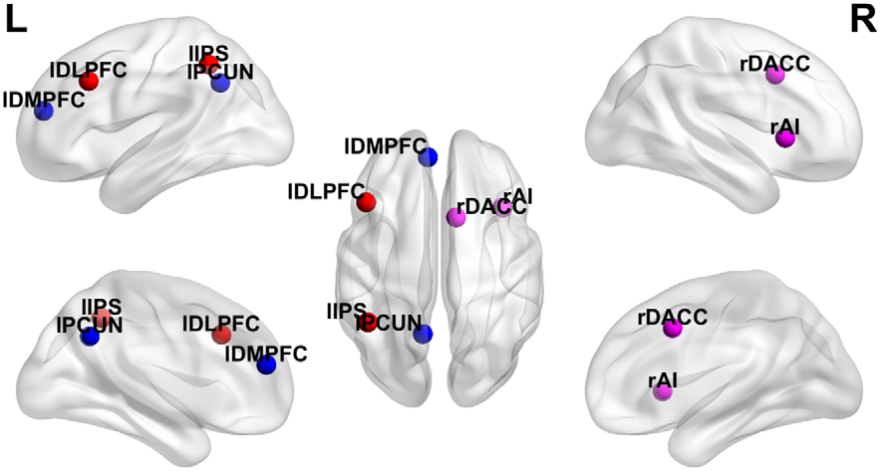
Regions of interest (ROIs) displayed using BrainNet Viewer (Xia, Wang, & He, 2013) on the ICBM-152 template brain (Mazziotta et al., 2001). Two ROIs in each of the executive control (red, lDLPFC: left dorsolateral prefrontal cortex and lIPS: left intra-parietal sulcus), default mode (blue, lDMPFC: left dorsomedial prefrontal cortex and lPCUN: left precuneus), and salience networks (pink, rAI: right anterior insula and rDACC: right dorsal anterior cingulate cortex). Figure 1. long description.

We extracted time series (first principal component) from each of six, 6-mm radius, spherical regions-of-interest (ROIs; Figure 1). Two ROIs represented ECN (left dorsolateral prefrontal cortex [DLPFC], centroid at *x* = −44, *y* = 22, *z* = 36; left intraparietal sulcus [IPS], *x* = −43, *y* = −50, *z* = 46; we focused on left-sided ECN nodes as the BRIGhTMIND trial targeted left DLPFC, given planned future analyses of changes in connectivity and cognition following rTMS). Two represented DMN (left dorsomedial prefrontal cortex [DMPFC], *x* = −7, *y* = 49, *z* = 18; left precuneus [PCUN], *x* = −10, *y* = −57, *z* = 35). Two represented SN (right anterior insula [AI], *x* = 38, *y* = 19, *z* = 2; right dorsal anterior cingulate cortex [DACC], *x* = 10, *y* = 13, *z* = 40; the SN, particularly its ECN-DMN switching function, is considered right dominant, e.g. Sridharan et al., 2008). The IPS, DMPFC, PCUN, and DACC centroids were taken from seeds used by Yeo et al. (2011) in their seven-network parcellation of the cortex. The DLPFC and AI centroids were taken from Fair et al. (2009) after conversion from Talairach to MNI152-space BioImage Suite (Papademetris et al., 2006). All centroids lay within the appropriate network on the Yeo et al. atlas, and the nearest node in the Power et al. (2011) atlas to each centroid was also assigned to the correct network.

After discarding the first five volumes and band-pass filtering (0.01–0.1 Hz), zero-lag Pearson correlations between time series from ROI pairs were computed, partialing 24 head motion parameters (current and prior six rigid-body parameters and their squares). Correlations were Fisher *r*-to-*z* transformed. Between-network connectivity was calculated as the mean of these values across pairs of ROIs.

### Prespecified statistical analyses

Analyses were conducted in StataNow SE 18.5 using multiple linear regression. Models included age, sex, MGH treatment-resistance group (low 2–3.5/medium 4–6/high 6.5+ as per trial cutoffs; Morriss et al., 2024), and site/scanner (six levels). Model diagnostics showed no need for additional transforms; variance inflation factors <5 indicated no multicollinearity concerns.

For the primary hypothesis, we tested whether DMN-ECN FC predicted IIV. DMN-ECN was the independent variable of interest. In separate analyses, MU, SIGMA, or TAU served as the dependent variable. Given the use of three dependent variables, false discovery rate correction [FDR] (Benjamini & Hochberg, 1995; Benjamini & Yekutieli, 2001) was applied across the three *p*-values for each predictor. Exploratory analyses used DMN-SN and SN-ECN FC as independent variables in place of, or alongside, DMN-ECN FC.

One secondary hypothesis concerned whether FC predicted subjective cognitive difficulties (PDQ-5-D). This was tested similarly, with PDQ-5-D as the single dependent variable. Another concerned whether DMN-ECN FC was predicted by SN-DMN and SN-ECN FC. DMN-ECN FC served as the dependent variable, and SN-DMN and SN-ECN FC were entered together as independent variables of interest. The final secondary hypothesis concerned whether the IIV measures predicted social and occupational functioning (WSAS). WSAS served as the dependent variable, and the three IIV variables were entered together as independent variables of interest.

### Synthesis of findings using path analysis

To synthesize findings from the primary and secondary analyses, we conducted a path analysis, using IBM SPSS Amos (v29). DMN-ECN and SN-ECN FC were allowed to predict the IIV variables (MU, SIGMA, and TAU) and functioning (WSAS). SN-ECN FC was also allowed to predict DMN-ECN FC. The IIV variables were allowed to predict functioning. Depression (HAMD) and anxiety (GAD-7) severity were considered exogenous and allowed to predict all other variables. The covariance between HAMD and GAD-7 was incorporated. All endogenous variables had error terms associated with them. Covariances between the error terms of the three IIV variables were included.

The model was fitted using maximum likelihood estimation. At the first stage, nonsignificant (*p* > 0.05) paths were removed, and the model was re-fitted. At the second stage, all nonsignificant paths and covariances were removed. The model was refitted and goodness-of-fit metrics examined (Supplementary Material 6). All paths and covariances were significant in the refitted model. Standardized and unstandardized regression weights were extracted and incorporated into a path diagram, alongside bias-corrected 90% confidence intervals derived from 10,000 bootstrap samples.

## Results

We examine each of the four prespecified hypotheses in turn, then synthesize findings across hypotheses using path analysis.

### Primary hypothesis: Relationship between IIV and FC between the DMN and ECN

**Figure 2.**
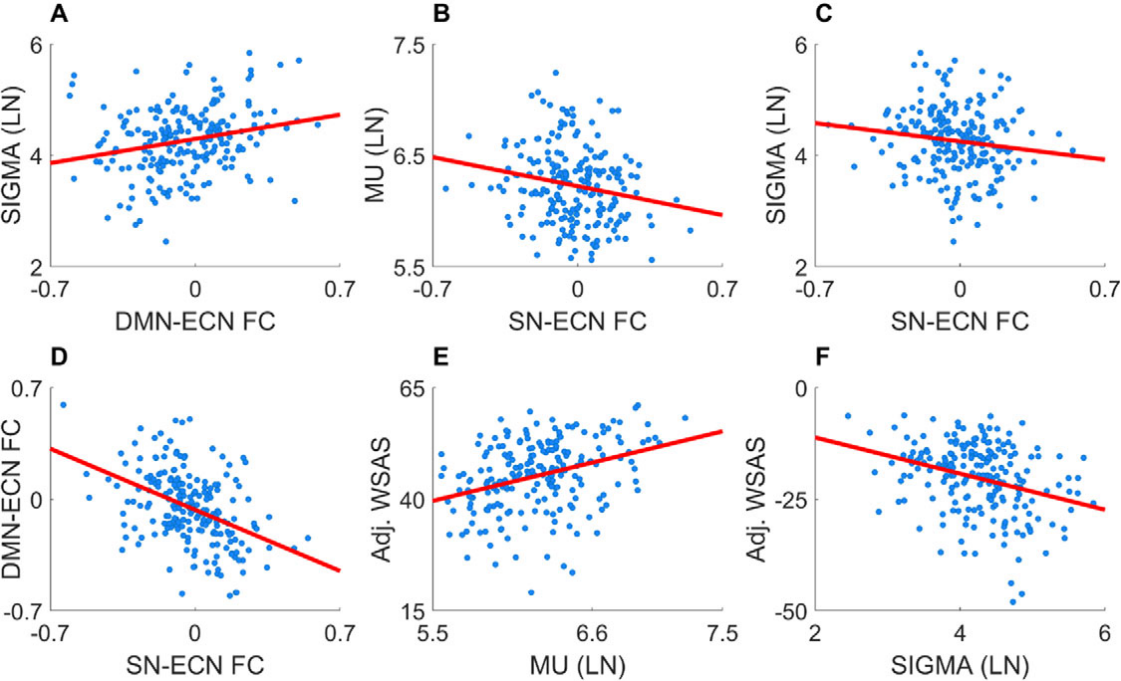
(a) SIGMA (standard deviation of the Gaussian component of reaction time distribution, higher values indicate greater reaction time variability) versus baseline DMN-ECN FC (each blue circle is a participant, trend line in red); (b) MU (mean of the Gaussian component reaction time distribution) versus SN-ECN FC; and (c) SIGMA versus SN-ECN FC. (d) DMN-ECN FC versus SN-ECN FC; (e) WSAS (functioning, higher scores indicate poorer functioning) versus MU (for display, the confounding influence of SIGMA has been regressed from WSAS scores); (f) WSAS versus SIGMA (for display, the confounding influence of MU has been regressed from WSAS scores). Figure 2. long description.

Our primary hypothesis was that greater intra-individual variability (IIV) on the Choice Reaction Time task would be associated with greater functional connectivity (FC) between the Default Mode Network (DMN) and the Executive Control Network (ECN). In multiple linear regressions, with each of the IIV variables (MU, SIGMA, and TAU) acting as the dependent variable in turn, and DMN-ECN FC as the predictor (alongside demographic/clinical variables), this hypothesis was supported. Greater (more positive) DMN-ECN FC was significantly associated with greater SIGMA (standard deviation of Gaussian component of reaction time distribution; Figure 2a), after false discovery rate (FDR) correction for the three analyses (*B* = 0.545, *β =* 0.204, *t* = 3.142, FDR-corrected *p* = 0.006; also see Supplementary Table S1). DMN-ECN FC was not a significant predictor of TAU (mean of exponential component of reaction time distribution; Supplementary Figure S2A) (*B* = -0.036, *β = −*0.010, *t* = −0.140, uncorrected *p* = 0.889). Greater DMN-ECN FC predicted greater MU (mean of Gaussian component; Supplementary Figure S2B) in uncorrected analyses only (*B* = 0.198, *β =* 0.131, *t* = 2.100, uncorrected *p* = 0.037, FDR-corrected *p* = 0.056). When incorporating HAMD and GAD-7 mood variables into the models, both relationships strengthened (SIGMA: *B* = 0.578, *β =* 0.217, *t* = 3.327, FDR-corrected *p* = 0.003; MU: *B* = 0.210, *β =* 0.139, *t* = 2.202, FDR-corrected *p* = 0.044; TAU: *B* = −0.044, *β = −*0.012, *t* = −0.172, uncorrected *p* = 0.863). The mood variables did not themselves predict MU or SIGMA (uncorrected *p* > 0.05). Greater HAMD predicted greater TAU in uncorrected analyses only (*B* = -0.029, *β =* 0.171, *t* = 2.082, uncorrected *p* = 0.039). See Supplementary Material 2 for pairwise analyses of individual DMN and ECN nodes. Findings were not impacted by global signal regression (Supplementary Material 3).

### Exploratory analysis of primary hypothesis: FC between other brain networks

We examined FC between the salience network (SN) and the DMN, and between the SN and the ECN, in place of FC between the DMN and ECN. None of the IIV variables were predicted by SN-DMN FC (Supplementary Figure S2C–E, uncorrected *p* > 0.05). However, greater (more positive) SN-ECN FC predicted lesser MU (Figure 2b) and SIGMA (Figure 2c; MU: *B* = –0.378, *β* = −0.198, *t* = 3.235, FDR-corrected *p* = 0.004; SIGMA: *B* = -0.493*, β* = −0.146, *t* = 2.236, FDR-corrected *p* = 0.040; TAU: Supplementary Figure S2F, *B* = 0.422, *β* = 0.092, *t* = 1.320, uncorrected *p* = 0.188). These relationships remained when incorporating mood variables into the models (MU: *B* = *−*0.379, *β* = −0.199, *t* = −3.237, FDR-corrected *p* = 0.004; SIGMA: *B* = −0.502*, β* = −0.149, *t* = −2.278, FDR-corrected *p* = 0.036). Examining MU and SIGMA with DMN-ECN and SN-ECN FC entered together as predictors, greater DMN-ECN FC predicted greater SIGMA (*B* = 0.461, *β* = 0.173, *t* = 2.398, FDR-corrected *p* = 0.035; MU: *B* = 0.083, *β* = 0.055, *t* = 0.806, uncorrected *p* = 0.421), while greater SN-ECN FC predicted lesser MU (*B* = −0.333, *β* = −0.174, *t* = −2.562, FDR-corrected *p* = 0.022; SIGMA: *B* = -0.241, *β* = −0.072, *t* = −0.998, uncorrected *p* = 0.320). Again, these relationships remained when incorporating mood variables into the models (DMN-ECN FC on SIGMA: *B* = 0.498, *β* = 0.187, *t* = 2.578, FDR-corrected *p* = 0.022; ECN-SN FC on MU: *B* = –0.328, *β* = −0.172, *t* = −2.515, FDR-corrected *p* = 0.026).

### Secondary hypothesis #1: Relationship between DMN-EC FC and subjective cognitive difficulties

We predicted that greater subjective/perceived cognitive difficulties (PDQ-5-D total score) would be associated with greater FC between the DMN and ECN. However, this was not supported (*p* > 0.05). In exploratory analyses, neither SN-DMN nor SN-ECN FC predicted PDQ-5-D.

### Secondary hypothesis #2: Relationship between DMN-ECN FC and SN-DMN or SN-ECN FC

In line with the view that the Salience Network controls connectivity between the Default Mode and Executive Control networks, we hypothesized that DMN-ECN FC would be predicted by both SN-DMN and SN-ECN FC. In multiple linear regression, with DMN-ECN FC as the dependent variable, and SN-DMN and SN-ECN FC entered together as predictors (alongside demographic/clinical variables), this hypothesis was partially supported.

Greater SN-ECN FC was associated with lesser DMN-ECN FC (Figure 2d; *B* = –0.546, *β* = −0.432, *t* = −6.754, *p* < 0.001). There was no relationship between SN-DMN FC and DMN-ECN FC (Supplementary Figure S2G, *B* = 0.008, *β* = 0.007, *t* = 0.115, *p* = 0.909). The relationship remained when incorporating mood variables into the model (*B* = -0.544, *β* = −0.430, *t* = −6.734, *p* < 0.001), and neither of the mood variables significantly predicted DMN-ECN FC (*p* > 0.05). See Supplementary Material 4 for pairwise analyses of individual network nodes. Findings were not impacted by global signal regression (Supplementary Material 5).

### Secondary hypothesis #3: Relationship between IIV and functioning

We predicted that greater IIV on the Choice Reaction Time task would be correlated with scores on the Work and Social Adjustment Scale (WSAS), a measure of social and occupational functioning. In multiple linear regression, with WSAS as the dependent variable, and the three IIV variables (MU, SIGMA, and TAU) entered together as predictors (alongside demographic/clinical variables), this hypothesis was supported. Greater mean reaction time (MU), lesser reaction time variability (SIGMA), and increased attentional lapses (TAU) were associated with poorer functioning (greater WSAS score) (MU: Figure 2e, *B* = 9.009, *β* = 0.405, *t* = 3.603, *p* < 0.001; SIGMA: Figure 2f, *B* = –3.713, *β* = −0.295, *t* = −2.747, *p* = 0.007; TAU: Supplementary Figure S2H, *B* = 1.639, *β* = 0.177, *t* = 2.522, *p* = 0.013). The relationship with TAU became nonsignificant when incorporating mood variables into the model (MU: *B* = 9.727, *β* = 0.438, *t* = 4.180, *p* < 0.001; SIGMA: *B* = -4.081, *β* = 0.259, *t* = −3.324, *p* = 0.002; TAU: *B* = 1.075, *β* = 0.116, *t* = 1.751, *p* = 0.082). Both greater HAMD and GAD-7 predicted poorer functioning in this model (HAMD: *B* = 0.338, *β* = −0.212, *t* = 2.777, *p* = 0.006; GAD-7: *B* = 0.370, *β* = 0.229, *t* = 3.164, *p* = 0.002).

### Path analysis linking mood, functional connectivity, IIV, and functioning

**Figure 3.**
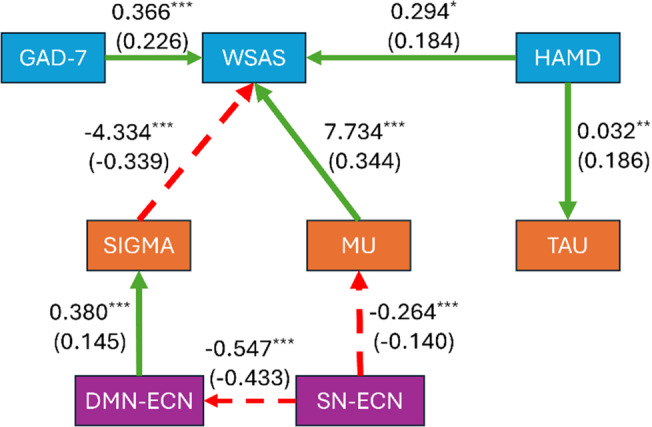
Final path model, showing relationships between DMN-ECN and SN-ECN FC, the three IIV variables (SIGMA, MU, and TAU), depression (HAMD), anxiety severity (GAD-7), and functioning (WSAS, higher scores indicate poorer functioning). Green solid arrows and red dashed arrows indicate positive and negative relationships, respectively. Values are regression weights, indicating the increment in the arrow’s target due to a one-unit increment in the arrow’s origin. Standardized regression weights are in brackets (increments in units of standard deviations). Covariances are omitted for visual clarity. *p < 0.05, **p < 0.01, and ***p < 0.005.

We conducted a path analysis to synthesize the above findings. We created a model in which each of the three IIV variables (MU, SIGMA, and TAU) could predict functioning (WSAS). DMN-ECN and SN-ECN FC could predict the IIV variables, as well as function directly. SN-ECN FC could predict DMN-ECN FC. Depression (HAMD) or anxiety (GAD-7) severity could predict the IIV variables, FC relationships, and functioning. An alternative approach of allowing the FC relationships to predict depression and anxiety severity was examined, but led to the same final model. Incorporating subjective cognitive difficulties (PDQ-5-D) into the model in a similar manner as the IIV variables led to significant paths from anxiety severity to subjective cognitive difficulties and from subjective cognitive difficulties to functioning; however, the structure of the rest of the model was unchanged. See Materials and Methods for details of model specification and fitting. The resulting model (Figure 3) met all examined goodness-of-fit indices (Supplementary Material 6, see also for covariance parameters).

Increased SN-ECN FC predicted faster average reaction times (lower MU; *B* = –0.264 [90% bias-corrected CI: −0.395 to −0.122], *β* = −0.140 [−0.211 to −0.063], *p* = 0.002) and lower DMN-ECN FC (*B* = −0.547 [−0.679 to −0.413], *β* = −0.433 [−0.523 to −0.333], *p* < 0.001). Increased DMN-ECN FC predicted greater reaction time variability (higher SIGMA; *B* = 0.380 [0.187–0.594], *β* = 0.145 [0.072–0.223], *p* = 0.002).

Greater depression severity (HAMD) predicted more frequent attentional lapses (higher TAU; *B* = 0.032 [0.013–0.050], *β* = 0.186 [0.075–0.289], *p* = 0.006) and poorer functioning (higher WSAS; *B* = 0.294 [0.111–0.486], *β* = 0.184 [0.070–0.299], *p* = 0.011). Greater anxiety severity (GAD-7) also predicted poorer functioning (*B* = 0.366 [0.167–0.575], *β =* 0.226 [0.104–0.353], *p* = 0.002).

Longer average reaction time (higher MU) predicted poorer functioning (higher WSAS; *B* = 7.734 [3.804–11.575], *β* = 0.344 [0.170–0.517], *p* < 0.001), while greater reaction time variability (higher SIGMA) predicted better functioning (lower WSAS; *B* = –4.334 [−6.549 to −2.054], *β* = −0.339 [−0.502 to −0.163], *p* < 0.001]. The indirect effect of DMN-ECN FC on functioning was significant (WSAS; *B* = –1.647 [−3.283 to −0.615], *β* = −0.049 [−0.094 to −0.019], *p =* 0.003), as was the indirect effect of SN-ECN FC on reaction time variability (SIGMA: *B* = –0.208 [−0.341 to −0.101], *β* = −0.063 [−0.105 to −0.030], *p* = 0.001; functioning, WSAS: *B* = −1.142 [−2.702 to −0.077], *β* = −0.027 [−0.064 to −0.002], *p* = 0.080).

## Discussion

We examined whether intra-individual variability (IIV) in attentional performance relates to large-scale network coupling in people with MDD. There are three key findings. First, in line with our primary hypothesis, greater coupling (FC) between the DMN and ECN was associated with greater reaction time variability (SIGMA, standard deviation of the Gaussian component of reaction time distributions). Second, greater SN–ECN FC predicted weaker DMN–ECN FC, as well as faster reaction times (MU, mean of the Gaussian component). Third, these properties of reaction time distributions (SIGMA and MU) predicted social and occupational functioning. We will also cover two additional points: the absence of relationships between network coupling and subjective cognitive difficulties, and the dependence of relationships between network coupling and attentional lapses (TAU, mean of exponential component) on mood severity.

In a meta-analysis of 26 resting-state FC datasets, Kaiser et al. (2015) found increased coupling between ECN and DMN regions in MDD (*N* = 556) compared to healthy controls (*N* = 518). This was confirmed in a later meta-analysis by Sha et al. (2019), incorporating 63 MDD datasets (*N* = 2023 patients, *N* = 1839 controls), which also found decreased coupling between the SN and ECN, and evidence of shared network changes across a range of psychiatric conditions. Here, we show that, in people with moderate–severe treatment-resistant MDD, both features are associated with cognitive performance, specifically reaction times on an attention-based task. Our data suggest that greater ECN-DMN FC drives up reaction time variability, whereas greater SN-ECN FC drives down average reaction time and exerts a suppressive influence on ECN-DMN FC.

The relationship between SN-ECN and ECN-DMN FC is consistent with the key role of the SN in inter-network regulation (Goulden et al., 2014; Sridharan et al., 2008). There is increasing evidence for the centrality of the SN to the pathophysiology of MDD (Lynch et al., 2024). The BRIGhTMIND trial itself found that connectivity between the (typically ECN) DLPFC target and the anterior insula (SN) predicted treatment outcomes (Morriss et al., 2024). Greater SN-ECN FC may lead to reduced average reaction times through greater ECN engagement and task focus.

We had speculated that increased ECN-DMN coupling may reflect intrusion of DMN-related internal world processing on ECN-related attention and task performance. While the relationship between increased ECN-DMN coupling and intra-individual reaction time variability appears to support this position, the finding that greater reaction time variability was associated with *better* social and occupational functioning in our sample necessitates reconsideration. Indeed, average reaction time and greater reaction time variability had mutually suppressive influences on functioning – better functioning was associated with lower average reaction time but greater reaction time variability. Methodologically, this underscores the importance of considering different components of reaction time distributions together in future studies.

It may be that prolonged average reaction time, with low reaction time variability, reflects a type of slowed, inflexible responding that is poorly suited to social and occupational success (in our sample of patients with long-standing, moderate–severe, treatment-resistant depression). Psychomotor slowing is known to strongly impact functioning in MDD (Papakostas, 2009; Porter, Bourke, & Gallagher, 2007). Relationships with reaction time variability are less well studied. It may be that reaction time variability, when distinguished from attentional lapses (themselves captured by the TAU parameter of the ex-Gaussian distribution), is a marker of cognitive flexibility, allowing one to improve performance and respond quickly when possible or wait for further certainty when necessary. There is evidence for cognitive rigidity and reduced adaptive control on attentional tasks in MDD (Meiran, Diamond, Toder, & Nemets, 2011; Stange et al., 2016; Zheng et al., 2024). Future research should use ex-Gaussian analyses or other distributional approaches that are able to disentangle contributors to reaction time variability (Parris, Dienes, & Hodgson, 2013; Viola et al., 2025) to further understand this phenomenon.

A key question is whether cognitive impairments are independent components of the pathophysiology of MDD or a secondary consequence of low mood (Bora, Harrison, Yücel, & Pantelis, 2013; Rock et al., 2014). Our observed relationships between DMN-ECN FC, SN-ECN FC, functioning, and average reaction time and variability of reaction time remained when depression and anxiety severity were considered, providing support for some degree of independence. However, relationships between FC or functioning and attentional lapses (TAU) were moderated by depression severity, and in the final path model, depression severity predicted TAU alone. There is some evidence for particular increases in TAU in people with depression versus healthy controls (Gallagher et al., 2015; Silvia et al., 2020). The current study examines relationships with reaction times within a sample of people with MDD, without comparison to healthy controls. Our findings nevertheless suggest that attentional lapses, in particular, would be expected to lessen as mood improves following a course of treatment.

We did not find relationships between the examined FC relationships and self-reported difficulties in cognition (as measured by the PDQ-5-D scale). When incorporated into the path model, subjective difficulties did predict functioning but were themselves predicted by anxiety symptoms. Serra-Blasco et al. (2019) have previously shown a discrepancy between objective and subjective cognition in MDD. It may be that subjective cognitive difficulties reflect perceived criticism of, or worry about, cognitive difficulties.

Key strengths of our study include the use of a pre-published analytic protocol to test a set of prespecified hypotheses based on the existing literature, which we synthesize into a well-fitting path model. We examine a large group of patients with generally long-standing, treatment-resistant depression of at least moderate severity, and we disentangle contributors to reaction time variability (Parris et al., 2013; Viola et al., 2025) using an ex-Gaussian model. Limitations of our study include that relationships between nonexamined brain networks or nodes may be missed. In particular, we prespecified fixed regions of interest (ROIs) to represent canonical brain networks, rather than identifying ECN, DMN, and SN sites or maps within individuals. We made this decision due to the expected increase in temporal dependency between the ECN and DMN in depression, which was the focus of the current analyses, and which could influence approaches to network identification that rely on statistical independence, such as independent component analysis (ICA). However, a fixed ROI approach has the disadvantage that it does not account for inter-individual variability in network topography (Marek & Dosenbach, 2018). Successful uses of ICA with dual regression in people with MDD (Li et al., 2017) suggest that such approaches could be explored with this dataset in the future. We also note our choice of left-sided ROIs to operationalize the ECN. This *a priori* decision reflected the use of a left DLPFC target (the most common neuromodulation target for MDD) in the BRIGhTMIND trial, given planned future analyses of changes in connectivity and cognition following neuromodulation. However, it should be noted that attentional brain networks are bilateral, with evidence for right-hemisphere dominance in some areas (Spagna, Kim, Wu, & Fan, 2020). Meta-analytic evidence supports a functional imbalance between left and right prefrontal cortices in MDD (Diener et al., 2012); thus, relationships between cognition and laterality of brain connectivity warrant further exploration.

Another limitation is that we examine functional (undirected) rather than effective (directed) brain connectivity metrics, so the causal influence between networks is less clear. The trial fMRI datasets used a long TR interval, unsuitable for analyses of effective connectivity. This, alongside short scan duration, also precluded time-varying (dynamic) functional connectivity analyses. Future work using dynamic connectome approaches may be valuable for understanding the immediate drivers of dysfunctions in attentional state changes (Song & Rosenberg, 2021). This will require the use of fMRI with concurrent tasks, as opposed to resting-state fMRI in the current study. Exploring IIV on a range of tasks, beyond the choice reaction time studied here, would help determine whether relationships generalize across tasks or reflect specific attention or psychomotor processes. Note also that, in the current study, the collection of imaging and task data could be separated by up to 2 weeks, so reported relationships reflect IIV and connectivity features that remain stable over this period in the depressed state.

As expected, given the patient group, most of those in the BRIGhTMIND trial were taking antidepressant medications, which were not stopped before trial commencement due to ethical concerns. Given the diversity of medications used, we could not directly explore medication effects in this study. It would be valuable to explore the impact of medication on relationships between brain connectivity, cognition, and functioning in future experimental studies. Finally, we treat functioning, measured with the Work and Social Adjustment Scale (WSAS) and incorporating work, close relationships, and private and social leisure activities, as a single dimension. There is evidence for a unidimensional factor structure for the WSAS, including in mental health conditions (Lundqvist et al., 2024; Zahra et al., 2014). However, it would be valuable to explore relationships between different aspects of functioning in future analyses. Our path model should be evaluated in future studies of people with moderate-to-severe treatment-resistant depression and both healthy and psychiatric controls (the latter to examine specificity to MDD; Sha et al., 2019).

In summary, we provide evidence for a model in which the SN, through coupling with the ECN, speeds up average reaction times and lowers coupling between the ECN and DMN. Greater ECN and DMN coupling predicts reaction time variability, and both reaction time variability and average reaction time predict functioning. Poorer functioning is associated with a type of slowed, inflexible responding. Our findings suggest that targeting the SN and seeking to enhance connectivity between the SN and ECN may provide a means of improving cognition and functioning in depression, and that such improvements might be expected to be separable from improvements in mood per se. It may be that targeting the relationship between the ECN and DMN directly would be counterproductive. Future analyses of the BRIGhTMIND dataset will examine the influence of TMS treatment on relationships between connectivity, reaction times, and functioning. This will provide further insights into treatment targets for cognitive difficulties, which are prevalent in MDD, often persist into euthymia, and are strong predictors of impairment.

## Supporting information

10.1017/S0033291726104899.sm001 Briley et al. supplementary material Briley et al. supplementary material

## Supplementary material

The supplementary material for this article can be found at http://doi.org/10.1017/S0033291726104899.

## Long descriptions

### Table 1. Long description

The table consists of four columns: Characteristic, Included n equals 209, Excluded n equals 46, and Comparison.

* Age in years mean S D: Included 44.4 14.0, Excluded 43.4 14.4. Comparison t 253 equals 0.444, p equals 0.658.

* Gender n percent: Men: Included 99 47.4 percent, Excluded 24 47.8 percent. Women: Included 110 52.6 percent, Excluded 22 52.2 percent. Comparison chi-squared 1 equals 0.349, p equals 0.555.

* Ethnicity n percent: White British: Included 176 84.2 percent, Excluded 38 82.6 percent. Other: Included 33 15.8 percent, Excluded 8 17.4 percent. Comparison chi-squared 1 equals 0.072, p equals 0.789.

* M G H treatment resistance category n percent: Low 2 to 3.5: Included 69 33.0 percent, Excluded 18 39.1 percent. Medium 4 to 6: Included 62 29.7 percent, Excluded 11 23.9 percent. High 6.5 plus: Included 78 37.3 percent, Excluded 17 37.0 percent. Comparison chi-squared 2 equals 0.850, p equals 0.654.

* H A M D total mean S D: Included 23.3 4.8, Excluded 24.4 4.7. Comparison t 253 equals 1.407, p equals 0.161.

* G A D 7 total mean S D: Included 13.3 4.7, Excluded 13.1 4.5. Comparison t 253 equals 0.287, p equals 0.775.

* W S A S total mean S D: Included 28.1 7.6, Excluded 29.2 6.2. Comparison t 253 equals 0.960, p equals 0.338.

Navigate back to Table 1..

### Figure 1. Long description

The visualization consists of five semi-transparent grey brain templates.

* Top-Left: Lateral view of the left hemisphere. A red sphere labeled l D L P F C is located anteriorly. A red sphere labeled l I P S and a blue sphere labeled l P C U N are located posteriorly.

* Bottom-Left: Medial view of the left hemisphere. A blue sphere labeled l D M P F C is at the anterior edge. A red sphere labeled l D L P F C is central. A blue sphere labeled l P C U N and a red sphere labeled l I P S are at the posterior edge.

* Center: Dorsal view showing both hemispheres. In the left hemisphere, l D M P F C is anterior, l D L P F C is lateral, and l I P S and l P C U N are posterior. In the right hemisphere, pink spheres r D A C C and r A I are located medially.

* Top-Right: Medial view of the right hemisphere. A pink sphere labeled r D A C C is superior and anterior. A pink sphere labeled r A I is inferior to it.

* Bottom-Right: Lateral view of the right hemisphere. The pink sphere r D A C C is visible in the superior frontal region, and r A I is visible in the insular region.

Color coding indicates functional networks: red for executive control, blue for default mode, and pink for salience networks.

Navigate back to Figure 1..

### Figure 2. Long description

A multi-panel set of six scatter plots labeled A through F. Each plot contains blue circular data points and a red linear trend line.

Panel A. X axis is D M N dash E C N F C from negative 0.7 to 0.7. Y axis is S I G M A L N from 2 to 6. The trend line shows a positive linear correlation.

Panel B. X axis is S N dash E C N F C from negative 0.7 to 0.7. Y axis is M U L N from 5.5 to 7.5. The trend line shows a negative linear correlation.

Panel C. X axis is S N dash E C N F C from negative 0.7 to 0.7. Y axis is S I G M A L N from 2 to 6. The trend line shows a slight negative linear correlation.

Panel D. X axis is S N dash E C N F C from negative 0.7 to 0.7. Y axis is D M N dash E C N F C from negative 0.7 to 0.7. The trend line shows a negative linear correlation.

Panel E. X axis is M U L N from 5.5 to 7.5. Y axis is Adj dot W S A S from 15 to 65. The trend line shows a positive linear correlation.

Panel F. X axis is S I G M A L N from 2 to 6. Y axis is Adj dot W S A S from negative 50 to 0. The trend line shows a negative linear correlation.

Navigate back to Figure 2..
