# Supplementary material for: Functional connectivity correlates of reaction time variability in treatment-resistant major depression
Source: Psychol Med. 2026 Jul 7;56:e217. doi: 10.1017/S0033291726104899 (PMC13370196; doi:10.1017/S0033291726104899)
Supplement: Briley et al. supplementary material [file S0033291726104899sup001.pdf]

SUPPLEMENTAL MATERIAL

Functional connectivity correlates of reaction time variability in treatment-resistant major depression

Paul M Briley, Lucy Webster, Beth Hall, Linda Davison, Peter Gallagher, Stefan Pszczolkowski, William J Cottam, Sudheer Lankappa, Dorothee P Auer, Peter F. Liddle[1], R. Hamish McAllister-Williams, Richard Morriss

Supplemental Figure S1

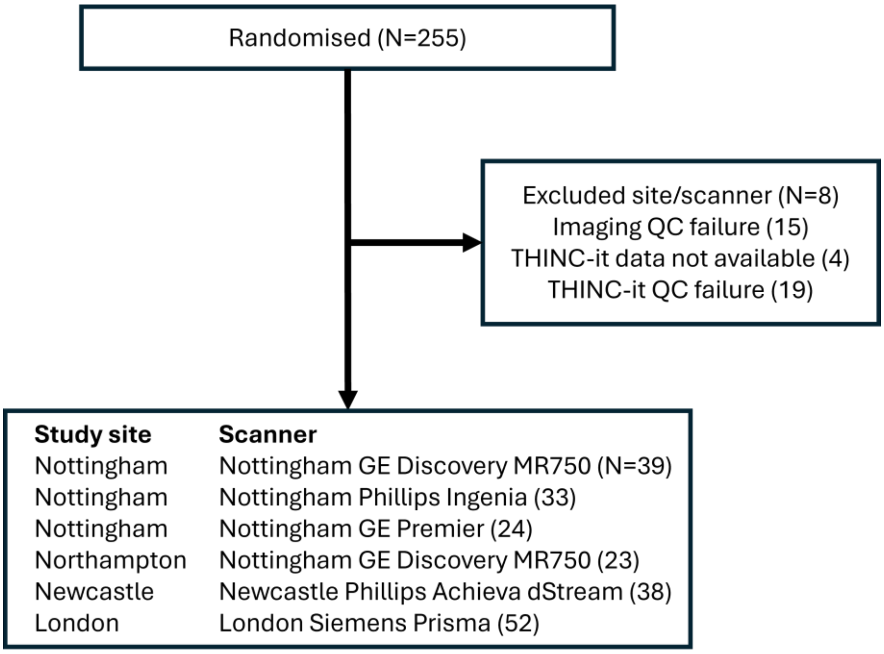

Flow chart illustrating reasons for exclusion from the current analyses (in line with the pre-specified cognition-imaging protocol) and number of participants from each combination of study site and scanner.

Supplemental Figure S2

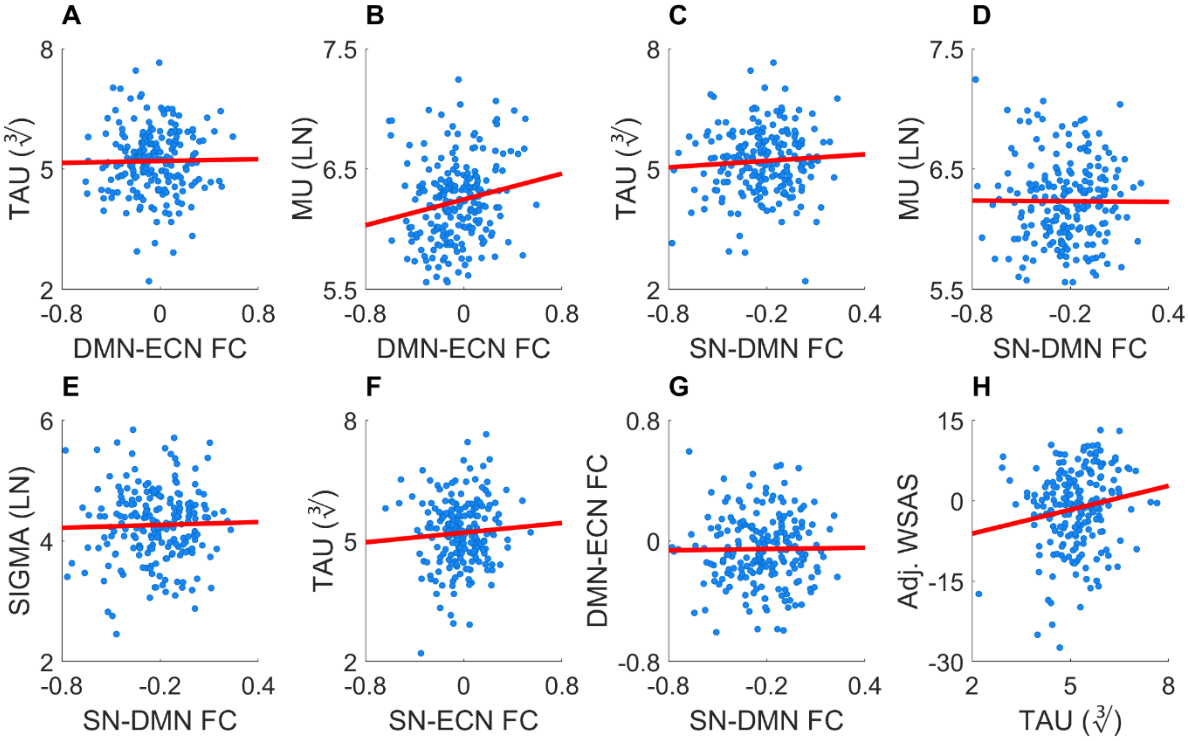

A: TAU (mean of exponential component of reaction time distribution) versus DMN-ECN FC (each blue circle is a participant, trend line in red); B: MU (mean of Gaussian component) versus DMN-ECN FC; C: TAU versus SN-DMN FC; D: MU versus SN-DMN FC; E: SIGMA (standard deviation of Gaussian component) versus SN-DMN FC; F: TAU versus SN-ECN FC; G: DMN-ECN FC versus SN-DMN FC; H: WSAS versus TAU (for display, the confounding influences of MU and SIGMA have been regressed from WSAS scores).

	MU	SIGMA	TAU	PDQ-5-D	DMN-ECN FC	WSAS
DMN-ECN FC	*B*=0.198<br>(0.012-0.384)<br>*β=0.131, t*=2.100<br>*p*=0.037<br>(FDR *p*=0.056)	*B*=0.545<br>(0.203-0.886)<br>*β=0.204, t*=3.142<br>*p*=0.002<br>(FDR *p*=0.006)**	*B*=-0.036<br>(-0.540-0.468)<br>*β=-0.010, t*=-0.140<br>*p*=0.889<br>(FDR *p*=1.000)	*B*=1.638<br>(-1.003-4.280)<br>*β*=0.092, *t*=1.224<br>*p*=0.223		
SN-DMN FC	*B*=-0.209<br>(-0.419-0.002)<br>*β=-0.122, t*=1.957<br>*p*=0.052<br>(FDR *p*=1.000)	*B*=-0.210<br>(-0.605-0.185)<br>*β=-0.070, t*=1.049<br>*p*=0.296<br>(FDR p=1.000)	*B*=0.167<br>(-0.403-0.736)<br>*β=0.041, t*=0.577<br>*p*=0.565<br>(FDR p=1.000)	*B*=1.623<br>(-1.343-4.589)<br>*β*=0.083, *t*=1.080<br>*p*=0.282	*B*=0.008<br>(-0.135-0.152)<br>*β*=0.007, *t*=0.115<br>*p*=0.909	
SN-ECN FC	*B*=-0.378<br>(-0.608- -0.148)<br>*β*=-0.198, *t*=3.235<br>*p*=0.001<br>(FDR *p*=0.004)***	*B*=-0.493<br>*(-0.927- -0.058)*<br>*β*=-0.146, *t*=2.236<br>*p*=0.027<br>(FDR *p*=0.040)*	*B*=0.422<br>(-0.208-1.053)<br>*β*=0.092, *t*=1.320<br>*p*=0.188<br>(FDR *p*=1.000)	*B*=-1.873<br>(-5.202-1.456)<br>*β*=-0.085, *t*=1.111<br>*p*=0.268	*B*=-0.546<br>(-0.705- -0.387)<br>*β*=-0.432, *t*=6.754<br>*p*<0.001***	
MU						*B*=9.009<br>(4.077-13.941)<br>*β*=0.405, *t*=3.603<br>*p*<0.001***
SIGMA						*B*=-3.713<br>(-6.378- -1.047)<br>*β*=-0.295, *t*=2.747<br>*p*=0.007**
TAU						*B*=1.639<br>(0.358-2.921)<br>*β*=0.177, *t*=2.522<br>*p*=0.013*

*Supplemental Table S1. Collated results for key linear regression analyses. For each cell, the row and column heading correspond to predictor and dependent variable, respectively. In line with the pre-specified analytic protocol, when MU, SIGMA, and TAU served as dependent variables, False Discovery Rate (FDR) correction was conducted across the three sets of analyses. When predicting DMN-ECN FC, SN-DMN/SN-ECN FC were entered together as predictors. When predicting WSAS, MU/SIGMA/TAU were entered together as predictors. All analyses also incorporated clinical/demographic factors as predictors (age, sex, MGH treatment-resistance group, and site/scanner). *p<0.05, **p<0.01, ***p<0.005 (refers to FDR-corrected values where relevant)*

Supplement 1: Relationships between FC and other THINC-measures

Cognitive findings in isolation have already been reported in Morriss et al. (2025). In our analysis protocol, we stated we would explore relationships between DMN-ECN, SN-ECN, and SN-DMN FC and performance on other THINC-it tasks: digit symbol substitution test (total number of correct responses); N-back task (total number of correct responses); and trail making task (log-transformed time to completion) (we did not have reaction time data for these tasks). We applied the same multiple linear regression approach as that reported for the choice reaction time task in the main text. There were no significant relationships between FC and performance on these other THINC-it tasks (uncorrected $p>0.05$).

Supplement 2: Relationship between DMN-ECN FC and IIV – pairwise analyses of individual network nodes

Considering the individual nodes of the DMN and ECN, the positive relationship with SIGMA was significant for the FC between left DLPFC (ECN) and both DMN nodes: left DMPFC [B=0.290, β=0.164, t=2.553, FDR-corrected p=0.034] and left precuneus [B=0.303, β=0.172, t=2.653, FDR-corrected p=0.026]. Relationships with SIGMA for FC between the left intra-parietal sulcus (IPS) ECN node and the DMN nodes did not reach significance [left IPS – left precuneus: B=0.225, β=0.120, t=1.802, uncorrected p=0.073; left IPS – left DMPFC: B=0.227, β=0.105, t=1.565, uncorrected p=0.119]. Positive relationships with MU did not reach significance for individual nodes [left DLPFC – left DMPFC: B=0.107, β=0.108, t=1.747, uncorrected p=0.082; left DLPFC – left precuneus: B=0.105, β=0.105, t=1.698, uncorrected p=0.091; left IPS – left precuneus: B=0.111, β=0.104, t=1.653, uncorrected p=0.100; left IPS – left DMPFC: B=0.048, β=0.039, t=0.614, uncorrected p=0.540].

Supplement 3: Impact of global signal regression on DMN-ECN findings

When global signal regression was incorporated into the analysis pipeline, DMN-ECN FC continued to be positively associated with SIGMA and MU [SIGMA: B=0.563, β=0.214, t=3.241, FDR-corrected p=0.004; MU: B=0.211, β=0.142, t=2.237, FDR-corrected p=0.040], and not TAU [B=-0.138, β=-0.039, t=-0.537, uncorrected p=0.592].

Supplement 4: Relationship between DMN-ECN FC and SN-ECN FC – pair-wise analyses of individual SN and ECN nodes

Considering individual network nodes, left DLPFC – left DMPFC FC was predicted by FC between left DLPFC and each of the SN nodes: right anterior insula (AI) [B=-0.460, β=-0.366, t=-5.343, p<0.001] and right dorsal anterior cingulate cortex (DACC) [B=-0.311, β=-0.235, t=-3.400, p<0.001]. Likewise, left DLPFC – left precuneus FC was predicted by FC between left DLPFC and each of the SN nodes [right AI: B=-0.786, β=-0.626, t=-10.860, p<0.001; right DACC: B=-0.695, β=-0.526, t=-8.781, p<0.001]. Similarly, left IPS – left DMPFC FC was predicted by FC between left IPS and each of the SN nodes [right AI: B=-0.417, β=-0.380, t=-5.936, p<0.001; right DACC: B=-0.290, β=-0.248, t=-3.677, p<0.001], as was left IPS – left precuneus FC [right AI: B=-0.623, β=-0.492, t=-8.108, p<0.001; right DACC: B=-0.565, β=-0.418, t=-6.572, p<0.001].

Supplement 5: Impact of global signal regression on SN-ECN findings

When global signal regression was incorporated into the analysis pipeline, DMN-ECN FC continued to be positively associated with SN-ECN FC [B=-0.632, β=-0.495, t=-8.206, p<0.001] and not with SN-DMN FC [B=-0.001, β=-0.001, t=-0.001, p=1.000].

Supplement 6: Goodness of fit statistics for path model and covariance parameters

Fit metrics included: the Chi-square test for discrepancy (a non-significant value suggests a close fit between the observed and model-implied covariance matrices); the ratio of the Chi-

square statistic to its degrees of freedom; the Comparative Fit Index (CFI) and Tucker-Lewis Index (TLI), which compare the Chi-square statistic to that of the null (independence) model while adjusting for sample size and model complexity; the Standardized Root Mean Square Residual (SRMR), which reflects the average standardised residual between observed and predicted correlations; and the Root Mean Square Error of Approximation (RMSEA), which quantifies the degree of approximation error in the population covariance matrix per degree of freedom, adjusting for model complexity.

The final path model described in the main text had nineteen parameters and met all examined goodness of fit indices. The chi-square test for discrepancy was non-significant, indicating adequate fit [$\chi2(17)=19.236$, $p=0.315$]. The ratio of the Chi-square statistic to its degrees of freedom was 1.132 (2.0 is a conservative upper limit indicating adequate fit; Tabachnick & Fidell, 2007). The Comparative Fit Index (CFI), and Tucker-Lewis Index (TLI), were 0.994, and 0.990, respectively (values > 0.95 indicate good model fit; Hu & Bentler, 1999; Hooper et al., 2008). The Standardised Root Mean Square Residual (SRMR) was 0.052, and the Root Mean Square Error of Approximation (RMSEA) was 0.025 (with 90% confidence interval: 0.000-0.070). SRMR < 0.07 and RMSEA < 0.06 (with upper confidence interval limit < 0.08), indicate good model fit (Hu & Bentler, 1999; Hooper et al., 2008). The covariance of anxiety and depression severity was significant [$\sigma x_{,y}$=11.184 (8.985 – 13.674), r=0.504 (0.423 – 0.579), p<0.001] as were the covariances between the error terms for SIGMA and MU [$\sigma x_{,y}$=0.150 (0.124 – 0.180), r=0.771 (0.714 – 0.815), p<0.001], and SIGMA and TAU [$\sigma x_{,y}$=-0.043 (-0.078- -0.012), r=-0.092 (-0.162- -0.025), p=0.029].

References

1. Hooper, D., Coughlan, J., & Mullen, M. (2008). Evaluating model fit: a synthesis of the structural equation modelling literature. In A. Brown (Ed.), *7th European Conference on Research Methodology for Business and Management Studies* (pp. 195-200).
2. Hu, L., & Bentler, P. M. (1999). Cutoff criteria for fit indexes in covariance structure analysis: conventional criteria versus new alternatives. *Structural Equation Modelling: A Multidisciplinary Journal, 6*(1), 1-55.
3. Morriss, R., Webster, L., Ingram, L., Abdelghani, M., Anton, A., Barber, S., ... Y., Auer, D. (2025). Connectivity guided intermittent theta burst stimulation versus repetitive transcranial magnetic stimulation in moderately severe treatment resistant depression: the BRIGhTMIND RCT. *Efficacy and Mechanism Evaluation, 12*(2).
4. Tabachnick, B. G., & Fidell, L. S. (2007). *Using Multivariate Statistics (5th ed.)*. New York: Allyn and Bacon.
